# Questioning the importance of LDL cholesterol: don’t throw the baby out with the bathwater!

**Published:** 2008-04

**Authors:** FJ Raal

**Affiliations:** Division of Endocrinology and Metabolism, Department of Medicine, Johannesburg Hospital, Johannesburg

Following the recent announcement of the disappointing results of the ENHANCE study,^1^ two press articles in the *Business Week* and the *New York Times* questioned the importance of lowering LDL cholesterol and asked ‘What’s cholesterol got to do with it’ and ‘Do cholesterol drugs do any good’.^2^,^3^ This resulted in a flurry of correspondence in the lay press questioning whether cholesterol has any role to play in the pathogenesis of atherosclerosis and coronary artery disease (CAD).

The ENHANCE study (Effect of Combination Ezetimibe and High-dose Simvastatin versus Simvastatin Alone on the Atherosclerotic Process in Patients with Heterozygous Familial Hypercholesterolaemia) was a study involving 720 subjects with heterozygous familial hypercholesterolaemia who were randomised to either simvastatin alone 80 mg daily, or a combination of simvastatin 80 mg plus ezetimibe 10 mg daily. The duration of the study was two years and the primary endpoint of the study was the mean change in carotid intima−media thickness (cIMT), a surrogate marker of atherosclerosis.

Despite a 17% greater reduction in LDL cholesterol with the combination therapy, there was no difference in the rate of progression of cIMT between the groups. There are several reasons for this unexpected result. Subjects were not statin naïve and had a baseline cIMT than was only minimally increased. More importantly, the mean LDL cholesterol level at baseline was 8.24 mmol/l and was reduced to only 4.86 mmol/l with simvastatin alone (a 41% reduction), and to 3.69 mmol/l (58% reduction) with the combination therapy. In order to achieve regression of atherosclerosis, the LDL cholesterol needs to be reduced to below 2.5 mmol/l, if not below 1.8 mmol/l, as was achieved in the REVERAL and ASTEROID studies.^4^,^5^ If a more potent statin had been used to reduce LDL cholesterol levels in the ENHANCE study, lower LDL-C levels would have been obtained and regression of cIMT may well have been seen.

Although there are several major risk factors for atherosclerosis, such as hypertension, diabetes and cigarette smoking, elevated LDL cholesterol is the driving force. Hunter-gatherer populations still following their indigenous lifestyle show little or no evidence of atherosclerosis, even in those individuals living into their seventh or eighth decades.^6^ In populations in whom serum cholesterol levels are low, such as the rural Chinese population, the prevalence of CAD is also low, even in the presence of other CAD risk factors. People with a genetic cause of lifelong low LDL cholesterol levels such as hypobetalipoproteinaemia or a mutation in PCSK9 have a markedly reduced risk of developing CAD despite a similar prevalence of other cardiovascular disease risk factors. In such subjects, the lifetime low LDL-C level lowers CAD risk by at least 80−90%.^7^

Innumerable epidemiological studies have shown a positive relationship between serum cholesterol levels and risk for CAD. In fact, the link between cholesterol and coronary artery disease is one of the most thoroughly researched and established facts in all of medicine.^8^ There is overwhelming evidence, accumulated over more than two decades, to show that the more you lower LDL cholesterol, the lower your risk. For every 1 mmol/l reduction in LDL-C using statins, the most powerful drugs we currently have for lowering LDL cholesterol levels, there is an approximately 12% reduction in total mortality and a 21% reduction in major vascular events.^9^ However, in large-scale lipid-lowering studies with the use of statins, overall reduction in events has been approximately 30%, with an average reduction in LDL cholesterol of 40%. There remains a ‘residual risk’ of approximately 60%. Why should this be? In my opinion it is a case of ‘too little, too late!’ The longer the LDL cholesterol is kept low and the lower the level achieved the greater the risk reduction.

Nearly every study has shown a reduction in mortality, particularity CAD mortality, with the use of statins. Three studies, however, the 4D study^10^ in subjects with advanced renal disease, the ASPEN study^11^ in type 2 diabetics, and the recently published CORONA study^12^ in subjects with cardiac failure did not show significant benefit. In these studies, atherosclerosis and the consequences thereof were already very advanced. One cannot expect statin therapy to ‘heal a broken heart’ or reverse end-stage renal disease. The important message from these negative studies is to treat earlier and more aggressively rather than to wait until atherosclerosis is advanced and irreversible damage has been done.

What about HDL cholesterol? In the recently published ILLUMINATE study, despite the very favourable lipid changes with the CETP inhibitor, torcetrapib, which increased HDL-C by 70%, the rate of cardiovascular events increased, rather than decreased by 25%.^13^ This may have been due to an unexplained toxic effect of the drug. However, until further outcome studies are available, the role of HDL-C elevation in reducing the cardiovascular event rate remains unproven.

Until the results of future studies using HDL-raising therapy are available, I would encourage you to reduce the LDL cholesterol as much and as early as possible in high-risk patients. Rather than questioning the lipid hypothesis, we should be treating earlier and more aggressively in those with diabetes, established vascular disease and familial hyperlipidaemia. Only then will we reduce the residual risk substantially. The LDL-C goal in such patients should be < 2.5 mmol/l, if not lower, if we want to reduce the event rate even further. These levels can often only be achieved with high doses of the more potent statins, such as rosuvastatin or atorvastatin, often in combination which other lipid-modifying drugs such as ezetimibe.

We have not yet identified a threshold below which LDL cholesterol reduction is no longer beneficial but harmful. We are born with an LDL cholesterol of 1 mmol/l, and it is probably only below this level that cholesterol synthesis will become limiting. If we can achieve this, perhaps the statement by Goldstein and Brown, the discoverers of the LDL receptor, ‘Heart attacks – gone with this century?’ may become a reality.^14^

Cardiovascular disease is, and probably will remain for several decades, the leading cause of death worldwide. LDL cholesterol is the pivotal risk factor. Having treated many patients with both homozygous and heterozygous familial hypercholesterolaemia for several years and having seen the remarkable benefits of statin therapy, I have a low threshold to treat patients with these remarkable, and remarkably safe drugs.^15^ Any sceptics who still need persuading should consider purchasing the book by Daniel Steinberg titled *The Cholesterol Wars. The Cholesterol Sceptics vs the Preponderance of Evidence*.^16^ Perhaps then they will change their minds.
